# 

**Published:** 1893-11

**Authors:** 


					﻿Vol XIII.
NOVEMBER, 1893.
NO. 11?*
THE
HOMEOPATHIC pHY^ICJlAp,
A Monthly Journal of Homoeopathic Materia Medica
and Clinical Medicine. •
"He is a freeman whom truth makes free, and all are slaves beside.”
"Seek the Truth: come whence it may, cost what it will.”
EDITED AND PUBLISHED BY
WALTER XI. JAMES, XI. D.
PHILADELPHIA :
No. 1125 SPRUCE STREET.
r'ON’DON :
ALFRED HEATH A CO., Sole Agents eor Great Britain,
114 Ebury Street, Eaton Square.
JKg~ Yearly Subscription, $2.50, invariably in advance. Single numbers, 25 Cts.
Entered *1 the Philadelphia PeaVOSe* a« Seeood-Clasa Mall Matter.
				

